# Phytochemicals of *Rhus* spp. as Potential Inhibitors of the SARS-CoV-2 Main Protease: Molecular Docking and Drug-Likeness Study

**DOI:** 10.1155/2021/8814890

**Published:** 2021-02-27

**Authors:** Yousery E. Sherif, Sami A. Gabr, Nasser M. Hosny, Ahmad H. Alghadir, Rayan Alansari

**Affiliations:** ^1^Clinical Pharmacology Department, Faculty of Medicine, Mansoura University, Mansoura, Egypt; ^2^Rehabilitation Research Chair, College of Applied Medical Sciences, King Saud University, Riyadh, Saudi Arabia; ^3^Department of Anatomy, Faculty of Medicine, Mansoura University, Mansoura, Egypt; ^4^Chemistry Department, Faculty of Science, Port Said University, Port Fuad, P O Box 42522, Egypt; ^5^Biology Department, Al-Ula Branch, Taibah University, Medina, Saudi Arabia

## Abstract

**Background:**

The outbreak of coronavirus disease 2019 (COVID-19) induced by the novel coronavirus severe acute respiratory syndrome coronavirus 2 (SARS-CoV-2) originated in China and spread to cover the entire world with an ongoing pandemic. The magnitude of the situation and the fast spread of the new and deadly virus, as well as the lack of specific treatment, led to a focus on research to discover new therapeutic agents.

**Aim:**

In this study, we explore the potential inhibitory effects of some active polyphenolic constituents of *Rhus* spp. (sumac) against the SARS-CoV-2 main protease enzyme (M^pro^; 6LU7).

**Methods:**

26 active polyphenolic compounds of *Rhus* spp. were studied for their antiviral activity by molecular docking, drug likeness, and synthetic accessibility score (SAS) as inhibitors against the SARS-CoV-2 M^pro^.

**Results:**

The results show that all tested compounds of sumac provided good interaction with the main active site of SARS-CoV-2 M^pro^, with better, lower molecular docking energy (kcal/mol) compared to the well-known drugs chloroquine and favipiravir (Avigan). Only six active polyphenolic compounds of *Rhus* spp. (sumac), methyl 3,4,5-trihydroxybenzoate, (Z)-1-(2,4-dihydroxyphenyl)-3-(3,4-dihydroxyphenyl)-2-hydroxyprop-2-en-1-one, (Z)-2-(3,4-dihydroxybenzylidene)-6-hydroxybenzofuran-3(2H)-one, 3,5,7-trihydroxy-2-(4-hydroxyphenyl)chroman-4-one, 2-(3,4-dihydroxyphenyl)-3,5-dihydroxy-7-methoxy-4H-chroman-4-one, and 3,7-dihydroxy-2-(4-hydroxyphenyl)chroman-4-one, were proposed by drug likeness, solubility in water, and SAS analysis as potential inhibitors of M^pro^ that may be used for the treatment of COVID-19.

**Conclusion:**

Six phenolic compounds of *Rhus* spp. are proposed for synthesis as potential inhibitors against M^pro^ and have potential for the treatment of COVID-19. These results encourage further in vitro and in vivo investigations of the proposed ligands and research on the preventive use of *Rhus* spp. against SARS-CoV-2.

## 1. Introduction

The coronavirus disease 2019 (COVID-19) crisis started in China in December 2019. By 30 January 2020, about 213 individuals had died and at least 9066 had been infected [1]. It also spread globally, first to a number of Asian countries, as well as to Canada, France, Germany, and the United States. As a result, due to the spread of this new and deadly virus, governments around the world put several major cities on lockdown and put aside all normal plans to deal with the crisis. In addition, on 30 January 2020, the World Health Organization (WHO) declared the COVID-19 outbreak a global health emergency because it could spread to countries that were not prepared [1–4]. Thus, on 11 March 2020, the WHO characterized COVID-19 as a pandemic, which has affected more than 200 countries; by March 2020, there were 30,105 deaths and 638,146 confirmed cases around the world [3], which have increased considerably over time. Genomic and molecular-based analyses show that SARS-CoV-2 is a new type of human-infected *β*-coronavirus (CoV), which suggested that zoonotic hosts like bats might be the original host of this virus [5]. Situation Reports released by the WHO on 13th April revealed that SARS-CoV-2 infection can induce severe collateral disorders such as severe pneumonia, pulmonary edema, acute respiratory disease syndrome (ARDS), or multiple organ failure (such as shock, acute heart injury, or acute kidney injury), which are ultimately responsible for an increased number of deaths worldwide [6]. This may be also due to the lack of an effective specific antiviral therapy, since SARS-CoV-2 is a novel pathogen. Thus, several drugs such as lopinavir/ritonavir, neuraminidase inhibitors, and other present antiviral drugs have been proposed for the treatment of COVID-19 infection [7, 8]. These drugs produce their antiviral potency via an inhibition process against SARS-CoV-2 proteases [7, 8]. The magnitude of the situation and the lack of a specific therapy for the virus have led to a focus on research to find new therapeutic agents [8]. For the management of COVID-19, preventive and supportive therapies based on herbal plants may complement research on existing antiviral agents.

Medicinal herbs known in ethnopharmacology have been suggested as antiviral agents for the treatment and control of contagious diseases like COVID-19 [9–14]. Most of these studies use molecular docking analysis to identify the potential activities of phytoconstituents present in herbal plants for the treatment of diseases like COVID-19. They focus on the activities of these plant constituents on the main proteases present in CoVs [12–14].

Sumac (*Rhus* spp.), a flowering plant that grows in temperate and tropical regions, contains over 250 individual species worldwide [15]. It is usually used as spice and a medicinal herb in most of the world, particularly for its antiviral [16, 17], antimicrobial, antibacterial, antioxidant, and wound-healing [18–26] properties.

The antiviral activity of *Rhus* spp., particularly *Rhus chinensis*, has been explored in many studies. Different phytochemical fractions of *Rhus chinensis* showed potent anti-HIV-1 [27–29], anti-herpes simplex virus (HSV) type 1 (HSV-1) [30–32], and anti-HCV activity. This antiviral activity was related to the presence of many active compounds such as phenolics, organic acids, proteins, fibers, volatile oils, fatty acids, vitamins, and minerals [33, 34]. Likewise, severe acute respiratory syndrome coronavirus (SARS-CoV) was significantly inhibited by using a 50% effective concentration (4.5 *μ*m) of tetra-O-galloyl-*β*-d-glucose isolated from Galla chinensis (*Rhus chinensis)* [35].

During infection, coronavirus attaches to target cells with the help of angiotensin-converting enzyme 2 (ACE2) present in the spike protein of the virus, which produces a spike protein-host cell protein interaction, whereby the virus genome with its nucleocapsid can easily release into the cytoplasm of the host cells [36, 37]. Sequence analysis of the replicase polyprotein in Avian infectious bronchitis virus, another coronavirus, originally predicted the presence of the coronavirus M^pro^ protease enzyme [38]. This enzyme was related to chymotrypsin-like cysteine proteases which significantly play a potential role in the replication and transcription of the coronavirus (SARS-CoV). Thus, it is considered a prime target for the discovery of antiviral agents [26, 39–41].

The SARS-CoV genome encodes a number of proteases. The main protease (M^pro^) chymotrypsin-like protease (3CL^pro^) from SARS-CoV-2 (6LU7) has an important role along with other cysteine proteases in the replication of the CoV genome. Thus, synthetic or herbal-based drugs targeting the proteases of SARS-CoV-2 (6LU7) may have a considerable role in the treatment of COVID-19 [38]. Several inhibitors including boceprevir, GC-376, and calpain inhibitors II and XII were identified to have potent activity to inhibit SARS-CoV-2 viral replication in cell culture [39]. The protease enzyme (6LU7) has been successfully crystallized and deposited in the Protein Data Bank (PDB) [40, 41]; thus, it is considered as a potential target for therapeutic strategies, particularly for those who use phytochemicals [7, 42, 43]. It was reported that an evaluation of up-to-date knowledge relating to the characteristics of COVID-19 infection and complications encourages the investigation of the effectiveness of sumac extracts for COVID-19 treatment [44–48]. Recently, active metabolites from 14 cooking seasonings were examined as inhibitors for SARS-CoV-2 main protease (M^pro^). A high potency of salvianolic acid A and curcumin as M^pro^ inhibitors with binding energies of −9.7 and −9.2 kcal/mol, respectively, was identified by in silico molecular docking analysis [47]; the potential activity of salvianolic acid A and curcumin as M^pro^ inhibitors against the SARS-CoV-2 main protease significantly depends upon forming from nine and six hydrogen bonds, respectively, with amino acids proximal to M^pro^'s active site [47]. Supporting review study showed that *Rhus* spp. (sumac) constituents could have a higher potency against the consequences produced by SARS-CoV-2 during human infection. The review study demonstrated that sumac could be useful in COVID-19 infection due to its versatile activities as anti-inflammatory, antimicrobial, antioxidant, and antimalarial effects [48]. In addition, the review article along with others mentioned that use of sumac as syrup or in capsules with different concentrations has no toxicity on human life and could be recommended in treatment protocol for COVID-19 patients [18, 48–51].

Regarding previous molecular docking studies [12, 18, 36, 44–52], we are trying to explore the potential inhibitory effects of some *Rhus* spp. (sumac) constituents against the SARS-CoV-2 main protease enzyme using molecular docking, drug likeness, and synthetic accessibility score analysis. The study will give more insight on the use of natural products as new therapeutic agents against the pathogenesis of the SARS-CoV-2 (COVID-19).

## 2. Methodology

### 2.1. *Rhus* spp. Phytoconstituents

**Figure 1:**
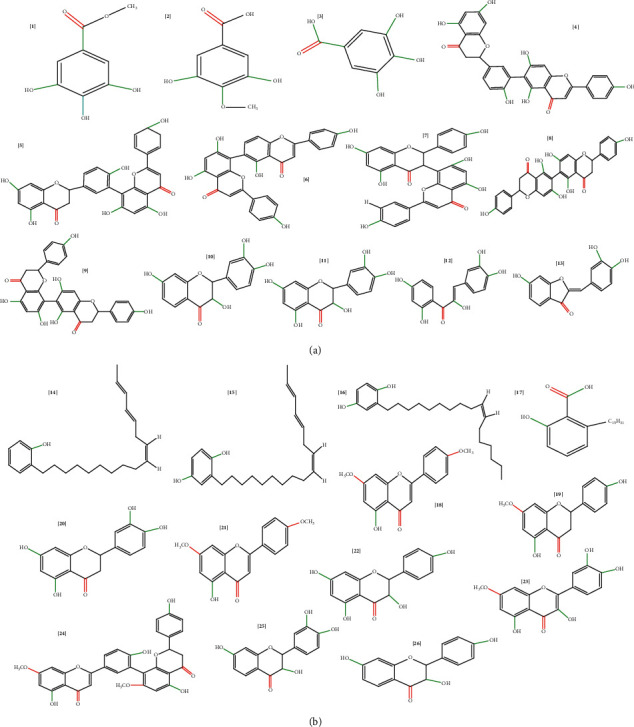
Chemical structures of 26 compounds (a and b) selected from the extract of *Rhus* spp. [44].

A total of 26 compounds previously extracted from *Rhus* spp. [52] were selected to study their potential activity against the SARS-CoV-2 main protease enzyme. These 26 components in *Rhus* spp. extracts (Figure 1) were selected based on their versatile biological activity, such as antimicrobial, antifungal, antidiabetic, antioxidant, wound-healing, and antiviral activity [26, 36, 52].

### 2.2. Molecular Docking Analysis

The crystal structure of the protease enzyme (the molecular target) of SARS-CoV-2 (3cl^pro^/M^pro^, PDB ID: 6LU7) was retrieved from RCSB Protein Data Bank (https://www.rcsb.org/) [53]. The proposed natural components in *Rhus* spp. extracts (Figure 1), in addition, well-known drugs, chloroquine and favipiravir (Avigan), widely tested in clinical trials for the treatment of COVID-19 have been subjected to molecular docking [54]. The structures of the selected sumac compounds along with the proposed drugs were drawn by ChemOffice (2015) freeware and were optimized by HyperChem v.8.1 to have the conformer with minimum energy using AMBER force field [54]. According to previously reported methodology and wording [14, 53–55], for analysis, an open Babel 2.3.2 software was used to convert a molecular format files into pdb format. Then, the ligands under investigations were loaded and their torsion along with rotatable bonds was assigned and saved as ligand PDBQT.

In our experiment by using Molegro Virtual Docker software 6.0, the binding modes of the proposed natural components in *Rhus* spp. extracts with the protease enzyme (the molecular target) of the SARS-CoV-2 (3cl^pro^/M^pro^; PDB ID: 6LU7) were identified [56]. In addition, a blind docking was performed to enumerate the strength of binding interactions between the proposed ligands and the protease enzyme target [14, 56]. In this step, a lowest binding energy (−kcal/mol) represents the best stable conformations of the ligand with the protease enzyme target [56]. Finally, according to the methodology previously reported [14], MGLTools-1.5.6 rc3 were used to analyze all released auto Dock output files (.dlg), whereas the docking parameters were represented as coordinates of the center of binding site with *x* = 126, *y* = 126, *z* = 126, and binding radius = 0.375 Ã. In addition, constituents that may hinder the simulation, such as water molecules, other heteroatoms, and native ligands attached to the target, were removed in the process of accommodating the ligands for the molecular docking study.

### 2.3. Drug-Likeness Analysis

This analysis qualitatively assesses the chance for a molecule to become an oral drug with respect to bioavailability, as previously described [57]. In this analysis, structural or physicochemical inspections of the proposed herbal compounds and selected drugs chloroquine and favipiravir (Avigan) were used to assess the drug likeness to be considered as an oral drug candidate. The analysis depends on five rule-based filters, with a diverse range of properties, inside of which the molecule is defined as drug-like. The Lipinski (Pfizer) filter is the pioneer rule-of-five implemented from [57]. The original rule-of-five (RO5) deals with orally active compounds and defines four simple physicochemical parameter ranges (MWT ≤ 500, log *P* ≤ 5, H-bond donors ≤ 5, and H-bond acceptors ≤ 10) associated with 90% of orally active drugs that have achieved phase II clinical status. These physicochemical parameters are associated with acceptable aqueous solubility and intestinal permeability and comprise the first steps in oral bioavailability [57–62]. This analysis is routinely performed to filtrate chemical libraries and exclude compounds with an incompatible or unacceptable pharmacokinetic profile.

In addition, the synthetic accessibility score (SAS) for each molecule extracted from sumac was calculated according to the methodology reported previously [57, 58]. SAS was shown to be essential in both the early drug discovery stage and the drug manufacturing process [59, 60]. To characterize the accessibility of drugs or molecules for synthesis, the synthetic accessibility score was grouped according to previous studies into three groups: easy to synthesize (SAS ≤ 3), moderately easy to synthesize (SAS = 3-4), and difficult to synthesize (>4) [57–63].

## 3. Results

### 3.1. Molecular Docking Analysis

The molecular docking parameters, including docking score, ligand-protein interactions, and hydrogen bonds for selected compounds and reference drugs, are provided in Table 1. The possible interactions with the active sites of protease enzyme in this experiment are discussed herein with some details. The reference drugs, Avigan and chloroquine, interact with active sites of protease enzyme 6LU7 with an energy docking score of −2.99 kcal/mol and −6.32 kcal/mol, respectively, as shown in Table 1.

All tested compounds of *Rhus* spp. have lower energy docking scores compared with the reference drugs Avigan and chloroquine (Table 1), as these compounds are polyphenols and can interact with 6LU7 more strongly through hydrogen bonds, pi-cation interactions, or pi-pi stacking interactions. Compound (**14**), although it demonstrated a good binding energy, could not form any hydrogen bond with the viral enzyme. Compound (**14**) showed no perceptible interactions but only electrostatics (Van der Waals) (see Supplementary file).

The obtained results show that the compounds methyl 3,4,5-trihydroxybenzoate (**1**), (Z)-1-(2,4-dihydroxyphenyl)-3-(3,4-dihydroxyphenyl)-2-hydroxyprop-2-en-1-one (12), (Z)-2-(3,4-dihydroxybenzylidene)-6-hydroxybenzofuran-3(2H)-one (13), 3,5,7-trihydroxy-2-(4-hydroxyphenyl)chroman-4-one (22), 2-(3,4-dihydroxyphenyl)-3,5-dihydroxy-7-methoxy-4H-chroman-4-one (23), and 3,7-dihydroxy-2-(4-hydroxyphenyl)chroman-4-one (26) give efficient interactions when complexed with the active sites of the protease enzyme 6LU7.

**Figure 2:**
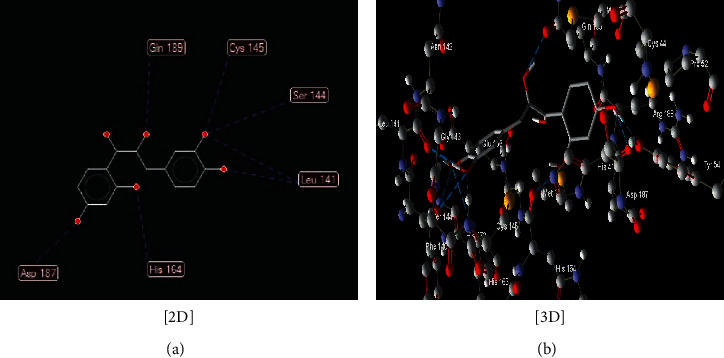
Interaction between compound (**12**) ((Z)-1-(2,4-dihydroxyphenyl)-3-(3,4-dihydroxyphenyl)-2-hydroxyprop-2-en-1-one) with COVID-19 (6LU7).

**Figure 3:**
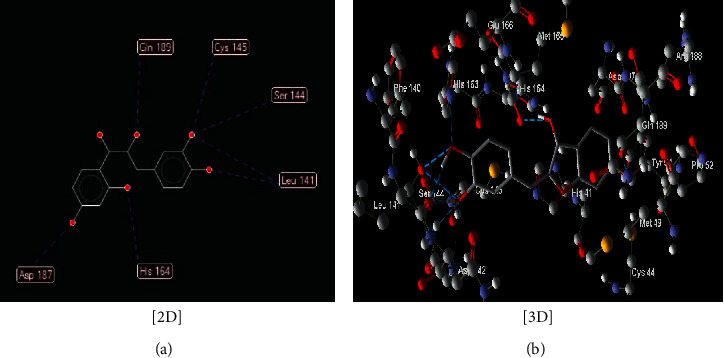
Interaction between compound (**13**) ((Z)-2-(3,4-dihydroxybenzylidene)-6-hydroxybenzofuran-3(2H)-one) with COVID-19 (6LU7).

**Figure 4:**
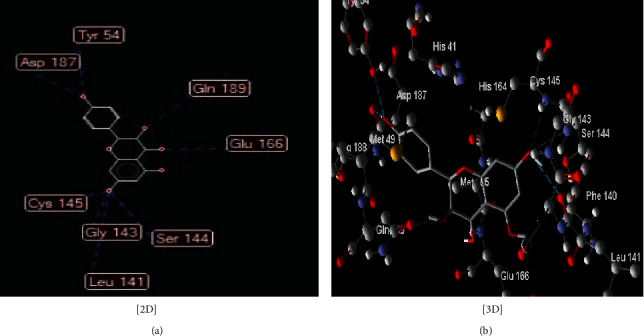
Interaction between compound (**22**) (3,5,7-trihydroxy-2-(4-hydroxyphenyl)chroman-4-one) with COVID-19 (6LU7).

**Figure 5:**
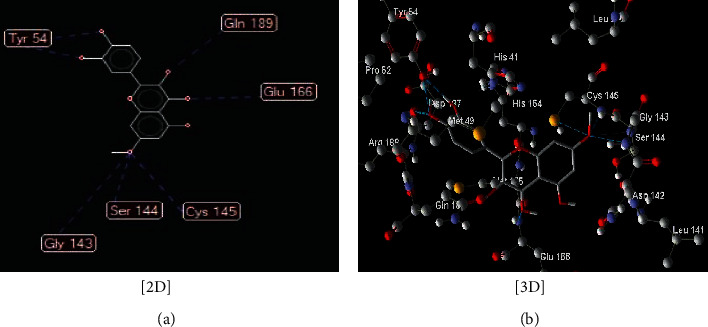
Interaction between compound (**23**) (2-(3,4-dihydroxyphenyl)-3,5-dihydroxy-7-methoxy-4H-chroman-4-one) with COVID-19 (6LU7).

**Figure 6:**
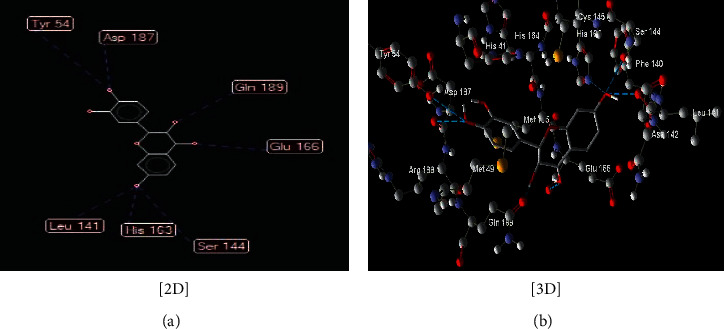
Interaction between compound (**26**) (3,7-dihydroxy-2-(4-hydroxyphenyl)chroman-4-one) with COVID-19 (6LU7).

**Figure 7:**
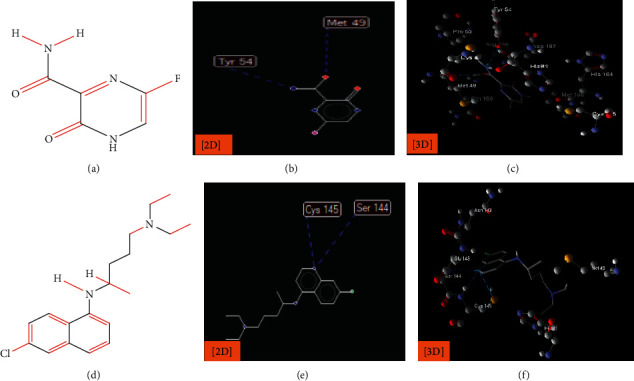
Interaction of the proposed drugs: favipiravir (Avigan): structure (a) and 2D and 3D structures (b and c); chloroquine: structure (d) and 3D structures (e and f) with COVID-19 (6LU7).

**Figure 8:**
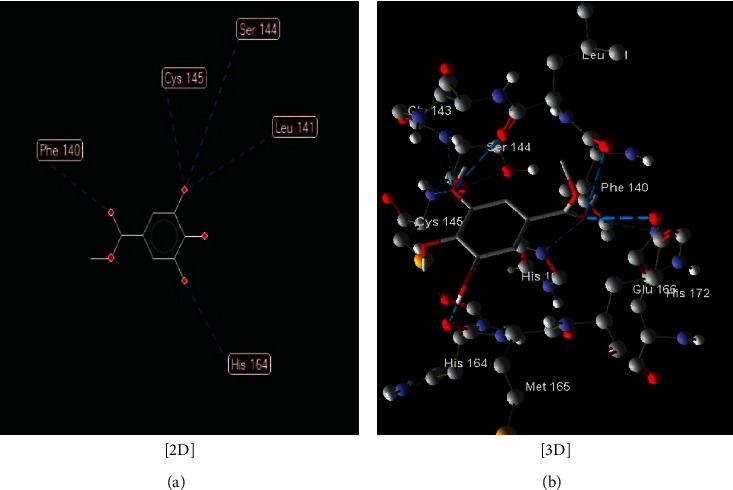
Interaction between compound (1) (methyl 3,4,5-trihydroxybenzoate) with COVID-19 (6LU7).

The interaction between these proposed compounds was efficient at lower energy docking scores, –22.6 kcal/mol for compound (1), −21.83 kcal/mol for compound (12), −14.31 kcal/mol for compound (13), −13.34 kcal/mol for compound (22), −15.57 kcal/mol for compound (23), and −17.21 kcal/mol for compound (26), as shown in Table 1. Interactions between proposed compounds (1), (12), (3), (22), (23), and (26) from *Rhus* spp. and 6LU7 are reported in Figures 2–7 and illustrated in Table 2. Also, the interaction of the proposed drugs, Avigan and chloroquine, with the active sites of protease 6LU7 of SARS-CoV-2 significantly proposed their potency for COVID-19 treatment as shown in Figure 8 and Table 2.

Due to the varied biological activity of the extracted polyphenolic compounds of *Rhus* spp. as potential active materials in the treatment of several diseases, several compounds are proposed: (1), (12), (13), (22), (23), and (26). These compounds showed the lowest docking energy with efficient interaction with the active sites of protease 6LU7 of SARS-CoV-2 through many hydrogen bonds compared with Avigan and chloroquine (Tables 1 and 2 and Figures 2–8).

### 3.2. Drug-Likeness Analysis

To study the availability of the proposed sumac compounds for synthesis, drug likeness, physicochemical properties, and synthetic accessibility score (SAS) were assessed according to the Lipinski rule-of-five. The drug likeness, water solubility, bioavailability scores, and SAS scores for all proposed active polyphenolic compounds of sumac and proposed Avigan and chloroquine are reported in Table 3.

The obtained results showed that six compounds (methyl 3,4,5-trihydroxybenzoate, (Z)-1-(2,4-dihydroxyphenyl)-3-(3,4-dihydroxyphenyl)-2-hydroxyprop-2-en-1-one, (Z)-2-(3,4-dihydroxybenzylidene)-6 hydroxybenzofuran-3(2H)-one, 3,5,7-trihydroxy-2-(4-hydroxyphenyl)chroman-4-one, 2-(3,4-dihydroxyphenyl)-3,5-dihydroxy-7-methoxy-4H-chroman-4-one, and 3,7-dihydroxy-2-(4-hydroxyphenyl)chroman-4-one) showed drug likeness with good solubility in water and higher bioavailability scores (0.55–0.56), and lower SAS range (1.5 to 3.42), as shown in Tables 3 and 4. These scores were efficient and optimal when compared to other docked compounds (4, 5, 6, 7, 8, 9, and 24) that showed no drug likeness (Table 3). This was due to the higher molecular weight of these compounds, which affects biological interactions and solubility in water, and consequently gave a lower bioavailability score (0.17) with a higher range of SAS (4.13 to 5.33), leading to difficulty in synthesizing them as drugs, as shown in Table 3. However, only six compounds (methyl 3,4,5-trihydroxybenzoate, (Z)-1-(2,4-dihydroxyphenyl)-3-(3,4-dihydroxyphenyl)-2-hydroxyprop-2-en-1-one, (Z)-2-(3,4-dihydroxybenzylidene)-6 hydroxybenzofuran-3(2H)-one, 3,5,7-trihydroxy-2-(4-hydroxyphenyl)chroman-4-one, 2-(3,4-dihydroxyphenyl)-3,5-dihydroxy-7-methoxy-4H-chroman-4-one, and 3,7-dihydroxy-2-(4-hydroxyphenyl)chroman-4-one) showed drug likeness with good solubility in water and higher bioavailability scores (0.55–0.56) and lower SAS range (1.5 to 3.42), as shown in Tables 3 and 4. The obtained physicochemical parameters, particularly higher bioavailability scores, good solubility, lower SAS, and drug likeness, in addition to the lowest energy docking score (kcal/mol), as shown in Table 4, argue for the use of these six compounds as potential inhibitors against the SARS-CoV-2 main protease enzyme.

## 4. Discussion

In this study, we are trying to decipher the proposed mechanism of the most potent active polyphenolic compounds extracted from *Rhus* spp. (sumac) in terms of binding affinity, necessary hydrogen bond formation [18, 54, 56], drug likeness, physicochemical properties, bioavailability, and synthetic accessibility score (SAS) [56–62], responsible for inhibition of target enzyme of the SARS-CoV-2. This study might lead to clinical trials for the treatment of infections caused by coronaviruses. Thus, a total of 26 known active polyphenolic compounds extracted from *Rhus* spp. (sumac) were measured for their activity as potential inhibitors against the SARS-CoV-2 main protease enzyme.

Molecular docking, drug-likeness analysis, and synthetic accessibility score (SAS) were performed for each active polyphenolic compound and compared with the results of Avigan and chloroquine as reference control drugs. All studied active polyphenolic compounds showed interactions with more hydrogen atoms to the main active site of the SARS-CoV-2 protease enzyme. This fits the complexation or interaction performed at lower energy docking score (kcal/mol) compared to that when Avigan and chloroquine interacted with the same active site. The polyphenolic nature of the extracted sumac compounds plays a pivotal role in their versatile biological activity, including antioxidant, antimicrobial, wound-healing, and antiviral activity [16–35, 64].

The presence of polyphenolic active groups with more hydrogen atoms in sumac compounds [57–60] easily facilitates the orientation and tight interaction with different amino acids present in the core of the main active site of the SARS-CoV-2 protease enzyme. In addition, it was reported previously that the antiviral activity of most herbal plants, including sumac, is related to the presence of active compounds such as phenolics, organic acids, proteins, fibers, volatile oils, fatty acids, vitamins, and minerals [33, 34].

To study the accessibility of the extracted active compounds of sumac for drug synthesis, drug-likeness analysis and SAS were performed. Only six active compounds (methyl 3,4,5-trihydroxybenzoate (1), (Z)-1-(2,4-dihydroxyphenyl)-3-(3,4-dihydroxyphenyl)-2-hydroxyprop-2-en-1-one (12), (Z)-2-(3,4-dihydroxybenzylidene)-6-hydroxybenzofuran-3(2H)-one (13), 3,5,7-trihydroxy-2-(4-hydroxyphenyl)chroman-4-one (22), 2-(3,4-dihydroxyphenyl)-3,5-dihydroxy-7-methoxy-4H-chroman-4-one (23), and 3,7-dihydroxy-2-(4-hydroxyphenyl)chroman-4-one (26)) showed a lower energy docking score (kcal/mol), acceptable with the Lipinski rule-of-five (drug likeness), good water solubility, high bioavailability (0.55–0.56), and lower range of SAS (1.5–3.42). These physicochemical parameters suggest the potential for synthesis as inhibitors against the main active site of the SARS-CoV-2 protease enzyme 6LU7. In our data, most of the proposed compounds, particularly, 12, 13, 23, and 26, along with Avigan and chloroquine, have hydrogen bonds with different lengths but identical bond energies, as shown in Table 2. This may be due to the fact that, in most proteins, almost all distances between two atoms are longer than the covalent bond length, so they tend to cause steric hindrance which may affect hydrogen bond energy during interaction with the main active site of the SARS-CoV-2 protease. In addition, all H atoms are donor rotatable, which could be sterically hindered by the surrounding atoms or molecules, thus resulting in the minimization of bond energies of H-bonds with longer lengths.

In most studies, the calculated SAS, drug likeness, bioavailability, water solubility, and lower energy docking scores (kcal/mol) could be used to support various drug discovery processes, particularly for herbal plants [54, 56–63]. It was reported previously that the drug discovery process is easier for ligands or compounds with lower SAS than higher SAS [38, 63, 65]. Like our results, ligands or compounds with the lowest energy docking scores, such as Nigellidine compound extracted from *Nigella sativa* L, when docked into the active site of 6LU7, produced lower energy with good orientation, which argues for its use as a potential drug candidate against COVID-19 [44]. Also, several molecular docking studies that used plant-based ligands such as kaempferol, quercetin, luteolin-7-glucoside, oleuropein, curcumin, catechin, epicatechin-gallate, caffeine, capsaicin, and hypericin show potential inhibitory activity against COVID-19 [9, 14, 38, 63, 65–68]. In addition, a high potency of salvianolic acid A and curcumin as herbal compounds were reported as M^pro^ inhibitors by using docking calculations [44].

## 5. Conclusion

The antiviral activity of 26 active polyphenolic compounds of *Rhus* spp. (sumac) was analyzed by molecular docking, drug likeness, and synthetic accessibility score (SAS). The results showed that all tested compounds provided good interaction in the main active site of the SARS-CoV-2 protease enzyme 6LU7 with lower molecular docking energy (kcal/mol) compared to two drugs already under clinical tests. Together, the molecular docking, drug likeness, and synthetic accessibility score (SAS) data proposed six active polyphenolic compounds of *Rhus* spp. for the synthesis of potential inhibitors against the main active site of the SARS-CoV-2 protease enzyme 6LU7, which may be candidates for the treatment of COVID-19. The data herein provide new directions for in vitro and in vivo investigations of the proposed ligands to develop new inhibitors as SARS-CoV-2 antivirals.

## Figures and Tables

**Table 1:** Physicochemical parameters and molecular docking scores of *Rhus* spp. (sumac) compounds obtained with 6LU7.

Compounds	Mol. dock scores (kcal/mol)	Protein ligand interactions	Hydrogen bonds energy (kcal/mol)	Properties
Favipiravir (Avigan)	−65.45	−75.47	−2.99	MW = 157.104; H-donor = 3; H-acceptor = 3

Chloroquine	−123.62	−135.43	−6.32	MW = 319.872; H-donor = 1; H-acceptor = 2

(1) Methyl 3,4,5-trihydroxybenzoate	−81.82	−72.2	−22.6	MW = 184.15; H-donor = 4; H-acceptor = 5

(2) 3,5-Dihydroxy-4-methoxybenzoic acid	−83.17	−75.33	−11.11	MW = 184.15; H-donor = 2; H-acceptor = 5

(3) 3,4,5-Trihydroxybenzoic acid	−73.23	−66.5	−12.02	MW = 170.12; H-donor = 3; H-acceptor = 5

(4) 6-(5-(5,7-Dihydroxy-4-oxochroman-2-yl)-2-hydroxyphenyl)-5,7-dihydroxy-2-(4-hydroxyphenyl)-4H-chromen-4-one	−135.36	−167.5	−13.5	MW = 540.47; H-donor = 8; H-acceptor = 10

(5) 8-(5-(5,7-Dihydroxy-4-oxochroman-2-yl)-2-hydroxyphenyl)-5,7-dihydroxy-2-(4 hydroxycyclohexa-1,5-dien-1-yl)-4H-chromen-4-one	−169.74	−194.15	−13.35	MW = 542.49; H-donor = 8; H-acceptor = 10

(6) 5,5′,7′-Trihydroxy-2,2′-bis(4-hydroxyphenyl)-4H,4′H-[6,8′-bichromene]-4,4′-dione	−150.98	−178.50	−12.79	MW = 522.46; H-donor = 7; H-acceptor = 9

(7) 8-(5,7-Dihydroxy-2-(4-hydroxyphenyl)-4-oxochroman-3-yl)-5,7-dihydroxy-2-(4-hydroxyphenyl)-4H-chromen-4-one	−155.04	−180.40	−13.26	MW = 540.47; H-donor = 8; H-acceptor = 10

(8) 5,5′,7,7′-Tetrahydroxy-2,2′-bis(4-hydroxyphenyl)-[6,6′-bichroman]-4,4′-dione	−151.40	−180.91	−14.47	MW = 542.49; H-donor = 8; H-acceptor = 10

(9) 5,5′,7,7′-Tetrahydroxy-2,2′-bis(4-hydroxyphenyl)-[6,8′-bichroman]-4,4′-dione	−146.88	−173.17	−1120	MW = 542.49; H-donor = 8; H-acceptor = 10

(10) 2-(3,4-Dihydroxyphenyl)-3,7-dihydroxychroman-4-one	−146.88	−173.17	−13.76	MW = 288.25; H-donor = 5; H-acceptor = 6

(11) 2-(3,4-Dihydroxyphenyl)-3,5,7-trihydroxychroman-4-one	−97.47	−121.77	−11.52	MW = 304.25; H-donor = 6; H-acceptor = 7

(12) (Z)-1-(2,4-Dihydroxyphenyl)-3-(3,4-dihydroxyphenyl)-2-hydroxyprop-2-en-1-one	−132.84	−142.30	−21.83	MW = 288.25; H-donor = 6; H-acceptor = 6

(13) (Z)-2-(3,4-Dihydroxybenzylidene)-6-hydroxybenzofuran-3(2H)-one	−125.38	−129.38	−14.31	MW = 270.24; H-donor = 4; H-acceptor = 5

(14) 2-((10Z,13E,15E)-Heptadeca-10,13,15-trien-1-yl)phenol	−93.31	−109.19	0.0	MW = 326.52; H-donor = 1; H-acceptor = 1–22

(15) 2-((10Z,13E,15E)-Heptadeca-10,13,15-trien-1-yl)benzene-1,4-diol	−141.68	−137.25	−5.39	MW = 342.51; H-donor = 2 H-acceptor = 2

(16) (Z)-2-(Heptadec-10-en-1-yl)benzene-1,4-diol	−131.14	−126.86	−4.54	MW = 346.55; H-donor = 2; H-acceptor = 2

(17) 2-Hydroxy-6 pentadecylbenzoic acid	−138.40	−134.16	−3.73	MW = 348.52; H-donor = 1; H-acceptor = 3

(18) 5-Hydroxy-7-methoxy-2-(4-methoxyphenyl)-4H-chromen-4-one	−116.41	−130.34	−5.51	MW = 298.29; -donor = 2; H-acceptor = 5

(19) 5-Hydroxy-2-(4-hydroxyphenyl)-7-methoxychroman-4-one	−107.68	123.39	−7.61	MW = 286.28; H-donor = 3; H-acceptor = 5

(20) 2-(3,4-Dihydroxyphenyl)-5,7-dihydroxychroman-4-one	−104.17	−121.38	−9.6	MW = 288.25; H-donor = 6; H-acceptor = 6

(21) 5-Hydroxy-7-methoxy-2-(4-methoxyphenyl)-4H-chromen-4-one	−114.79	−129.56	−6.3	MW = 298.29; H-donor = 2; H-acceptor = 5

(22) 3,5,7-Trihydroxy-2-(4-hydroxyphenyl)chroman-4-one	−109.41	−130.17	−13.34	MW = 288.25; H-donor = 5; H-acceptor = 6

(23) 2-(3,4-Dihydroxyphenyl)-3,5-dihydroxy-7-methoxy-4H-chromen-4-one	−105.80	−125.70	−15.57	MW = 316.26; H-donor = 5; H-acceptor = 7

(24) 5-Hydroxy-2-(4-hydroxy-3-(5-hydroxy-2-(4-hydroxyphenyl)-7-methoxy-4-oxochroman-8-yl)phenyl)-7-methoxy-4H-chromen-4-one	−179.18	−189.69	−13.35	MW = 568.53; H-donor = 6; H-acceptor = 10

(25) 2-(3,4-Dihydroxyphenyl)-3,7-dihydroxychroman-4-one	−106.43	−115.60	−8.57	MW = 288.25; H-donor = 5; H-acceptor = 6

(26) 3,7-Dihydroxy-2-(4-hydroxyphenyl)chroman-4-one	−107.14	−123.05	−17.21	MW = 272.25; H-donor = 4; H-acceptor = 5

**Table 2:** Interactions between the sites of 6LU7 in complex with the main proposed *Rhus* spp. (sumac) compounds for COVID-19 treatment.

Compound	Name	Type of interactions
Drugs	Favipiravir (Avigan) (Figure 8)	Hydrogen interactions are possible with the following:
		Amino acid Tyr 54 (H-donor rotatable) with distance about 2.67 Å and energy of −2.5 kcal/mol
		Amino acid Met 49 (H-donor rotatable) with distance about 2.91 Å and energy of −2.5 kcal/mol
	Chloroquine (Figure 8)	Hydrogen interactions are possible with the following: Amino acid Cys 145 by (two H-donors) with distance about 3.15 and 3.02 Å and energy of −2.24, 1.33 kcal/mol Amino acid Ser 144 (H-donor) with distance about 3.12 Å and energy of −1.13 k cal/mol


1	Methyl 3,4,5-trihydroxybenzoate (Figure 2)	Five hydrogen interactions are possible with the following:
		Amino acid Phe 140 (H-donor rotatable) with distance about 3.07 Å and energy of −2.50 kcal/mol
		Amino acid Leu 141 (H-donor rotatable) with distance about 3.11 Å and energy of −2.42 kcal/mol
		Amino acid Ser 144 (H-donor rotatable) with distance about 3.13 Å and energy of −2.13 kcal/mol
		Amino acid Cys 145 (H-donor rotatable) with distance about 3.2.96 Å and energy of −1.26 kcal/mol
		Amino acid His 164 (H-donor rotatable) with distance about 3.18 Å and energy of −2.08 kcal/mol

12	(Z)-1-(2,4-Dihydroxyphenyl)-3-(3,4-dihydroxyphenyl)-2-hydroxyprop-2-en-1-one (Figure 3)	Seven hydrogen interactions are possible with the following:
		Amino acid Gln 189 (H-donor rotatable) with distance 2.71 Å and energy of −2.5 kcal/mol
		Amino acid Cys 145 (H-donor rotatable) with distance 3.08 Å and energy of −2.5 kcal/mol
		Amino acid Leu 141 (H-donor rotatable) with distance 3.02 Å and energy of −2.5 kcal/mol
		Aminoa Leu 141 (H-donor rotatable) with distance 2.93 Å and energy of −2.5 kcal/mol
		Amino acid Ser 144 (H-donor rotatable) with distance 3.11 Å and energy of −2.44 kcal/mol
		Amino acid His 164 (H-donor rotatable) with distance 2.64 Å and energy of −2.50 kcal/mol
		Amino acid Asp 187 (H-donor rotatable) with distance 3.21 Å and energy of −1.97 kcal/mol

13	(Z)-2-(3,4-Dihydroxybenzylidene)-6-hydroxybenzofuran-3(2H)-one (Figure 4)	Seven hydrogen interactions are possible with the following:
		Amino acid His 164 (H-donor rotatable) with distance 2.88 Å and energy of −2.5 kcal/mol
		Amino acid Leu 141 (H-donor rotatable) with distance 2.87 Å and energy of −2.50 kcal/mol
		Amino acid Leu 141 (H-donor rotatable) with distance 2.62 Å and energy of −2.50 kcal/mol
		Amino acid Cys 145 (H-donor) with distance 3.10 Å and energy of −1.28 kcal/mol
		Amino acid Ser 144 (H-donor rotatable) with distance 2.91 Å and energy of −2.50 kcal/mol
		Amino acid Gln 189 (H-donor rotatable) with distance 2.71 Å and energy of −2.5 kcal/mol
		Amino acid Asp 187 (H-donor rotatable) with distance 3.21 Å and energy of −1.97 kcal/mol

22	3,5,7-Trihydroxy-2-(4-hydroxyphenyl)chroman-4-one (Figure 5)	Eight hydrogen interactions are possible with the following:
		Amino acid Asp 187 (H-donor rotatable) with distance 3.17 Å and energy of −2.15 kcal/mol
		Amino acid Tyr 54 (H-donor rotatable) with distance 2.89 Å and energy of −2.50 kcal/mol
		Amino acid Gln 189 (H-donor rotatable) with distance 2.59 Å and energy of −2.39 kcal/mol
		Amino acid Ser 144 (H-acceptor) with distance 2.90 Å and energy of −1.21 kcal/mol
		Amino acid Leu 141 (H-donor rotatable) with distance 3.11 Å and energy of −2.45 kcal/mol
		Amino acid Gly 143 (H-acceptor) with distance 3.16 Å and energy of −0.83 kcal/mol
		Amino acid Cys 145 (H-donor rotatable) with distance 3.00 Å and energy of −1.47 kcal/mol
		Amino acid Glu 166 (H-acceptor) with distance 2.96 Å and energy of −2.33 kcal/mol

23	2-(3,4-Dihydroxyphenyl)-3,5-dihydroxy-7-methoxy-4H-chroman-4-one (Figure 6)	Eight hydrogen interactions are possible with the following:
		Amino acid Tyr 54 (H-donor rotatable) with distance 2.66 Å and energy of −2.5 kcal/mol
		Amino acid Tyr 54 (H-donor rotatable) with distance 2.93 Å and energy of −2.5 kcal/mol
		Amino acid Cys 145 (H-donor rotatable) with distance 3.30 Å and energy of −1.50 kcal/mol
		Amino acid Cys 145 (H-donor rotatable) with distance 3.08 Å and energy of −2.41 kcal/mol
		Amino acid Gln 189 (H-donor rotatable) with distance 3.29 Å and energy of −1.53 kcal/mol
		Amino acid Glu 166 (H-acceptor) with distance 2.96 Å and energy of −2.33 kcal/mol Amino acid Ser 144 (H-donor rotatable) with distance 3.09 Å and energy of −0.92 kcal/mol
		Amino acid Gly 143 (H-donor rotatable) with distance 2.86 Å and energy of −1.38 kcal/mol

26	3,7-Dihydroxy-2-(4-hydroxyphenyl)chroman-4-one (Figure 7)	Seven hydrogen interactions are possible with the following:
		Amino acid Tyr 54 (H-donor rotatable) with distance 2.67 Å and energy of −2.5 kcal/mol
		Amino acid Asp 187 (H-donor rotatable) with distance 2.79 Å and energy of −2.5 kcal/mol
		Amino acid Gln 189 (H-donor rotatable) with distance 2.5897 Å and energy of −2.5 kcal/mol
		Amino acid Glu 166 (H-donor rotatable) with distance 3.11 Å and energy of −2.40 kcal/mol
		Amino acid Ser 144 (H-donor rotatable) with distance 2.64 Å and energy of −2.50 kcal/mol
		Amino acid His 163 (H-donor rotatable) with distance 3.15 Å and energy of −2.59 kcal/mol
		Amino acid Leu 141 (H-donor rotatable) with distance 2. 93 Å and energy of −2.50 kcal/mol

**Table 3:** Physicochemical parameters and drug-likeness scores of *Rhus* spp. (sumac) compounds obtained with 6LU7.

Compounds	Mol. w. **(**g/mol)	Water solubility	Drug likeness	Bio. score	Synthetic accessibility score
Drugs					
Favipiravir (Avigan)	157.104	Very soluble	Yes	0.55	2.03
Chloroquine	319.872	Moderately soluble		0.55	2.76
(1) Methyl 3,4,5-trihydroxybenzoate	184.15	Very soluble	Yes	0.55	1.5
(2) 3,5-Dihydroxy-4-methoxybenzoic acid	184.15	Very soluble	Yes	0.56	1.38
(3) 3,4,5-trihydroxybenzoic acid	170.12	Very soluble	Yes	0.56	1.22
(4) 6-(5-(5,7-Dihydroxy-4-oxochroman-2-yl)-2-hydroxyphenyl)-5,7-dihydroxy-2-(4-hydroxyphenyl)-4H-chromen-4-one	540.47	Poorly soluble	No	0.17	4.60
(5) 8-(5-(5,7-Dihydroxy-4-oxochroman-2-yl)-2-hydroxyphenyl)-5,7-dihydroxy-2-(4 hydroxycyclohexa-1,5-dien-1-yl)-4H-chromen-4-one	542.49	Moderately soluble	No	0.17	5.33
(6) 5,5′,7′-Trihydroxy-2,2′-bis(4-hydroxyphenyl)-4H,4′H-[6,8′-bichromene]-4,4′-dione	522.46	Poorly soluble	No	0.17	4.13
(7) 8-(5,7-Dihydroxy-2-(4-hydroxyphenyl)-4-oxochroman-3-yl)-5,7-dihydroxy-2-(4-hydroxyphenyl)-4H-chromen-4-one	540.47	Poorly soluble	No	0.17	4.98
(8) 5,5′,7,7′-Tetrahydroxy-2,2′-bis(4-hydroxyphenyl)-[6,6′-bichroman]-4,4′-dione	542.49	Poorly soluble	No	0.17	4.58
(9) 5,5′,7,7′-Tetrahydroxy-2,2′-bis(4-hydroxyphenyl)-[6,8′-bichroman]-4,4′-dione	542.49	Poorly soluble	No	0.17	4.66
(10) 2-(3,4-Dihydroxyphenyl)-3,7-dihydroxychroman-4-one	288.25	Soluble	Yes	0.55	3.46
(11. 2-(3,4-Dihydroxyphenyl)-3,5,7-trihydroxychroman-4-one	304.25	Soluble	Yes	0.55	3.51
(12) (Z)-1-(2,4-Dihydroxyphenyl)-3-(3,4-dihydroxyphenyl)-2-hydroxyprop-2-en-1-one	288.25	Moderately soluble	Yes	0.55	2.78
(13) (Z)-2-(3,4-Dihydroxybenzylidene)-6-hydroxybenzofuran-3(2H)-one	270.24	Moderately soluble	Yes	0.55	2.89
(14) 2-((10Z,13E,15E)-Heptadeca-10,13,15-trien-1-yl)phenol	326.52	Poorly soluble	Yes	0.56	3.35
(15) 2-((10Z,13E,15E)-Heptadeca-10,13,15-trien-1-yl)benzene-1,4-diol	342.51	Poorly soluble	Yes	0.55	3.46
(16) (Z)-2-(Heptadec-10-en-1-yl)benzene-1,4-diol	346.55	Poorly soluble	Yes	0.55	3.23
(17) 2-Hydroxy-6 pentadecylbenzoic acid	348.52	Poorly soluble	Yes	0.56	2.97
(18) 5-Hydroxy-7-methoxy-2-(4-methoxyphenyl)-4H-chromen-4-one	298.29	Moderately soluble	Yes	0.55	3.14
(19) 5-Hydroxy-2-(4-hydroxyphenyl)-7-methoxychroman-4-one	286.28	Soluble	Yes	0.55	3.11
(20) 2-(3,4-Dihydroxyphenyl)-5,7-dihydroxychroman-4-one	288.25	Soluble	Yes	0.55	3.11
(21) 5-Hydroxy-7-methoxy-2-(4-methoxyphenyl)-4H-chromen-4-one	298.29	Moderately soluble	Yes	0.55	3.14
(22) 3,5,7-Trihydroxy-2-(4-hydroxyphenyl)chroman-4-one	288.25	Soluble	Yes	0.55	3.42
(23) 2-(3,4-Dihydroxyphenyl)-3,5-dihydroxy-7-methoxy-4H-chromen-4-one	316.26	Soluble	Yes	0.55	3.30
(24) 5-Hydroxy-2-(4-hydroxy-3-(5-hydroxy-2-(4-hydroxyphenyl)-7-methoxy-4-oxochroman-8-yl)phenyl)-7-methoxy-4H-chromen-4-one	568.53 g/mol	Poorly soluble	No	0.17	4.93
(25) 2-(3,4-Dihydroxyphenyl)-3,7-dihydroxychroman-4-one	288.25	Soluble	Yes	0.55	3.46
(26) 3,7-Dihydroxy-2-(4-hydroxyphenyl)chroman-4-one	272.25	Soluble	Yes	0.55	3.36

**Table 4:** Chemical structures, physicochemical properties, and energy docking scores (kcal/mol) of the main proposed *Rhus* spp. (sumac) compounds for COVID-19 treatment.

Compound	Name	Hydrogen bond energy (kcal/mol)	Drug-likeness properties			
			Drug likeness	Water solubility	Bio. score	Synthetic accessibility score
1	Methyl 3,4,5-trihydroxybenzoate	−22.6	Yes	Very soluble	0.56	1.5
12	Z)-1-(2,4-Dihydroxyphenyl)-3-(3,4-dihydroxyphenyl)-2-hydroxyprop-2-en-1-one	−21.83	Yes	Moderately soluble	0.55	2.78
13	(Z)-2-(3,4-Dihydroxybenzylidene)-6-hydroxybenzofuran-3(2H)-one	−14.31	Yes	Moderately soluble	0.55	2.89
22	3,5,7-Trihydroxy-2-(4-hydroxyphenyl)chroman-4-one	−13.34	Yes	Soluble	0.55	3.42
23	2-(3,4-Dihydroxyphenyl)-3,5-dihydroxy-7-methoxy-4H- chroman-4-one	−15.57	Yes	Soluble	0.55	3.30
26	3,7-Dihydroxy-2-(4-hydroxyphenyl)chroman-4-one	−17.21	Yes	Soluble	0.56	3.36

## Data Availability

The data used to support the findings of this study are available from the corresponding author upon request.

## Supplementary Materials

Supplementary Materials Interaction between compound (**14**) (2-((10Z,13 E,15E)-heptadeca-10,13,15-trien-1-yl)) phenol with COVID-19 (6LU7). The 2- and 3-dimensional (2D and 3D) structures of compound (**14**) showed no hydrogen bonding and no perceptible interactions, only electrostatics exist (Van der Waals).
