# Supplementary material for: Phytochemicals of Rhus spp. as Potential Inhibitors of the SARS-CoV-2 Main Protease: Molecular Docking and Drug-Likeness Study
Source: Evid Based Complement Alternat Med. 2021 Feb 27;2021:8814890. doi: 10.1155/2021/8814890 (PMC7937479; doi:10.1155/2021/8814890)
Supplement: Supplementary Materials — Interaction between compound (14) (2-((10Z,13 E,15E)-heptadeca-10,13,15-trien-1-yl)) phenol with COVID-19 (6LU7). The 2- and 3-dimensional (2D and 3D) structures of compound (14) showed no hydrogen bonding and no perceptible interactions, only electrostatics exist (Van der Waals). [file 8814890.f1.docx]

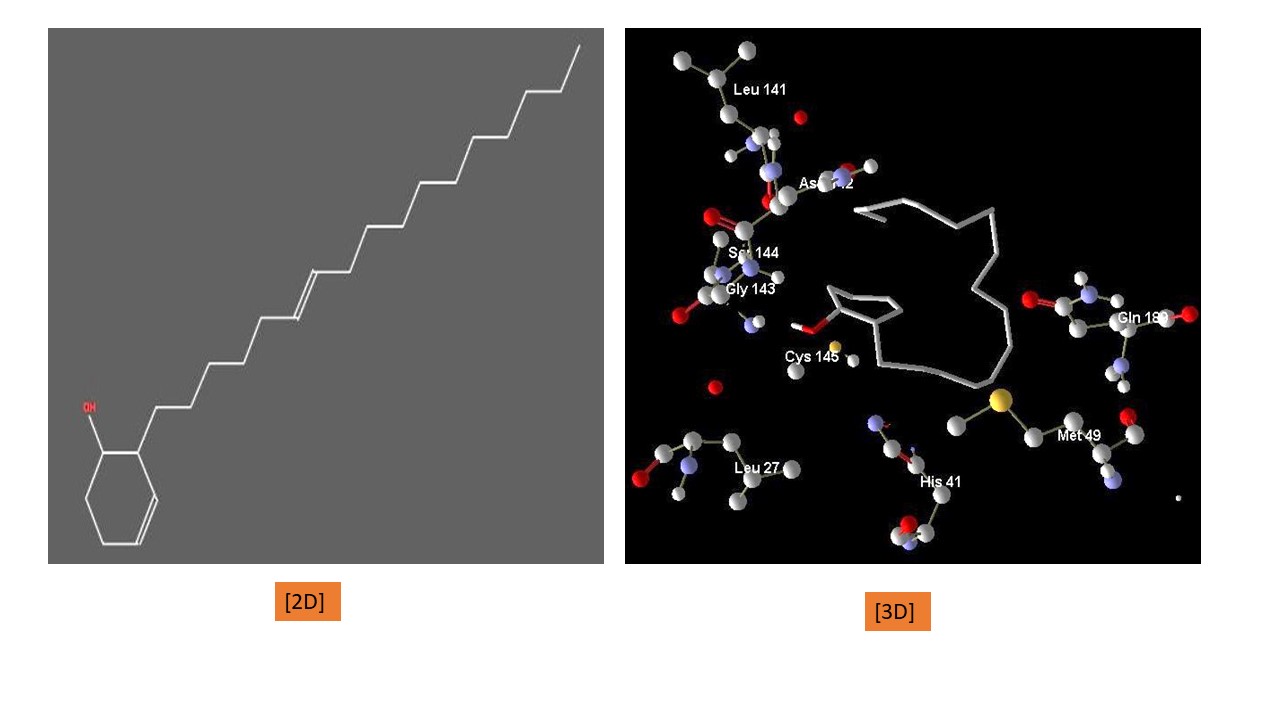


Interaction between compound [14]; 2-((10Z,13E,15E)-heptadeca-10,13,15-trien-1-yl) phenol with COVID-19 (6LU7). The 2- and 3-dimensional (3D) structures of compound [14] showed no hydrogen bonding and no perceptible interactions, only electrostatic exist (Van der Waals).
